# Non-invasive wool hormone assessment of Australian merino rams (*Ovis aries*): a pilot investigation of cortisol and testosterone

**DOI:** 10.3389/fvets.2024.1448232

**Published:** 2024-08-19

**Authors:** Dylan Fox, Benn Wilson, Edward Narayan

**Affiliations:** School of Agriculture and Food Sustainability, Faculty of Science, The University of Queensland, Gatton, QLD, Australia

**Keywords:** stress, non-invasive biomarkers, HPA axis, fibre, reproduction

## Abstract

**Introduction:**

Non-invasive hormone assessment is growing in interest as producers and livestock researchers seek new methods to assess animal welfare. Non-invasive wool assessment offers long-term, historic reflections of hormone concentration at the scale of weeks and months - and are not limited by sampling stress - thus making wool an appropriate tissue for long-term hormone analysis. This pilot study quantified cortisol and testosterone concentrations of ram fleece and determined if there is a significant difference between segments of the sample staple, and whether there is a correlation between hormones. Cortisol is a glucocorticoid produced within the adrenal glands and secreted in anticipation of or in response to a stressor. Testosterone is an androgen mainly synthesised within the testes of males and responsible for several critical functions including regulation of muscle growth, libido and spermatogenesis.

**Methods:**

In our study, 70 topknot wool samples were collected from rams on a commercial stud property in Dirranbandi, Queensland, Australia. Of these animals, 12 samples were selected at random to undergo cortisol and testosterone quantification. In the laboratory, a single, intact staple was isolated from the total sample, divided into 10 mm segments and prepared for their respective (cortisol or testosterone) immunoassays.

**Results:**

No significant difference (*p* > 0.05) was found between wool segments for either cortisol or testosterone, however, statistical differences (*p* < 0.05) were found between individuals for both hormones. A strong positive correlation (R^2^ = 0.9173, *p* < 0.05) was found between wool cortisol and testosterone concentrations.

**Discussion:**

In summary, this study reveals the major future possibilities for non-invasive wool hormone assessment in merino rams.

## Introduction

The stress response is a natural reaction to the perception of a threat and initially attempts to maintain homeostasis despite the stressor (1, 2). Stress can have significant effects on livestock (3). Cortisol is a steroid hormone in the class of glucocorticoids, colloquially referred to as the ‘stress hormone’ for its role in the physiological stress response. Cortisol analysis provides an indication of activation of the hypothalamo–pituitary–adrenal (HPA) axis, which mediates the stress response, with support from the sympatho–adrenal–medullary (SAM) axis (4). Cortisol actively circulates the body via blood and is found in numerous bodily fluids, including saliva, excreta (urine and faeces), hair, and wool (5).

Testosterone, an androgen and the primary male sex hormone, is essential for the normal maturation of male animals because it promotes several critical developments, including protein synthesis, which contributes to the greater size of males, increased bone density, and proper penis, scrotal, and testes development (6). It is also necessary for spermiogenesis in male animals. In seasonal breeders, including the ram, reproductive axis activation occurs in relation to day length (7). Declining daylight stimulates oestrus behavior in ewes and elevated testosterone production in males (8). Testosterone is fundamentally important for libido and continual sexual activity by rams. Early research on growth rate and feed utilisation efficiency found that ram lambs outperformed their castrated counterparts and attributed the difference to testosterone (9–11) recognised that sexual activity increases with increase in testosterone levels.

Wool hormone analysis is a non-invasive method useful for retrospective studies due to the delay in hormone incorporation into wool (12, 13). Hair or wool has been used as a better alternative to blood samples for long-term hormone assessment (4, 14–18). Circulatory steroids are gradually integrated into the hair shaft whilst it grows, and as such, analysing the hair or wool of the animal can give a long-term record of the changes in steroids, such as cortisol, in the animal (4, 15–19). Steroids incorporated in the hair/wool shaft are not affected by the daily fluctuations of the hormones and can store the steroid changes for weeks or even months, depending on the species (4, 15–19). A study by Nejad et al. (15) that used both blood and wool samples in order to measure cortisol in sheep under heat stress and water restrictions concluded that cortisol in wool was a more accurate method of measuring stress in the animals used (15, 17).

The study objective is to evaluate whether there is a statistically significant difference between wool cortisol and testosterone across the length of the staple. It is hypothesised that no statistically significant difference will be found within the staple for each hormone.

## Methods

### Ethics approval

Biological samples were obtained with the University of Queensland ethics approval (with protocol approval number 2021/AE000485).

### Fieldwork

#### Animal husbandry

All 70 rams (2 years of age) used in this study were owned by Wilgunya Merino Stud, located in Dirranbandi, Queensland, 4,486 (−28.856123236319117, 148.4624339024027). At the time of our visit (14 October 2022), all rams were housed in a separate paddock with *ad libitum* access to natural grasslands, shade trees, and fresh drinking water. Property managers used all terrain vehicles (ATVs) and sheep dogs to herd the rams into the race for sample collection.

#### Wool collection

Rams were run through the race in batches of 20 rams, where an identifying tag number was noted on both a resealable bag and a strip of paper, which was placed within the bag. Handheld electric clippers were used to recover a sample of topknot fleece (all rams were sampled at the same site on the mid forehead portion on top of the crown), which was placed within its own labelled bag. Rams were restricted in the race for an average of 10 min between sampling of the first and last ram. A total of 70 samples of ram fleece were collected. A wool sample was placed in a brown paper bag (labelled with ram ID and date) and kept in a plastic container at room temperature. All samples were collected by the farm support worker to reduce the risk of animal injury by inexperienced personnel. Upon return to the University of Queensland, Gatton Campus, 4,343 (−27.55331668592056, 152.3344294537852), all ram fleece samples were placed into a large vacuum-sealable bag and placed within a Waeco freezer at −20°C.

## Laboratory analysis

### Wool preparation

The methodology used was adapted from the study by Sawyer et al. (19). Before use, the vacuum-sealed bag was reinflated and allowed to reach room temperature before samples were catalogued. Of the 70 samples, a random selection (by handpicking) of 12 rams was made for this study. The main reason was due to the limited resources available to process and analyse all 70 samples (This could provide over 500 sub-samples in total). From each of these 12 ram wool samples, an intact, clean, unstretched staple was identified and gently removed from the fleece by hand. This piece was deemed the representative sub-sample for that individual moving forward. All other samples were returned to the freezer.

Using a ruler marked with 1 mm increments, each sub-sample was cut into 10-mm pieces [representing monthly wool growth in sheep; (12)] and assigned to a labelled weighing boat. The samples were labelled as “animal ID number” and a letter, ranging from A to G. For instance, 4C represented the third piece from ram #4. Sample “A” was furthest to the scalp, and “G” was the closest. The number of 10-mm sub-samples varied between rams due to differences in total staple length. The weight of all samples—minus the weighing boat weight—was recorded using a digital balance (Ohaus Pioneer™) accurate to three decimal places. To avoid human error, the balance was connected to a laptop via an RS-232 cable to allow for the direct transmission of weight data into Microsoft Excel.

### Washing procedure

Early findings by Davenport et al. (4) using hair and subsequent studies using wool recommended the use of isopropanol as a washing agent to remove external contamination from the sample surface (19). As such, each 10-mm sample taken from the 12 wool sub-samples was submerged in 3 mL of 100% isopropanol for 5 min, drained, and placed within a glass desiccator until dry. Each 10-mm sample was typically dried within 48 h of washing and was then diced finely using forceps and surgical scissors and added to an Eppendorf tube with 1 mL of 100% ethanol for hormone extraction. The Eppendorf tubes were briefly vortexed for at least 1 s to ensure maximum ethanol penetration of the finely cut wool. The tubes were labelled and placed in a refrigerator.

### Cortisol assay

**Figure 1:**
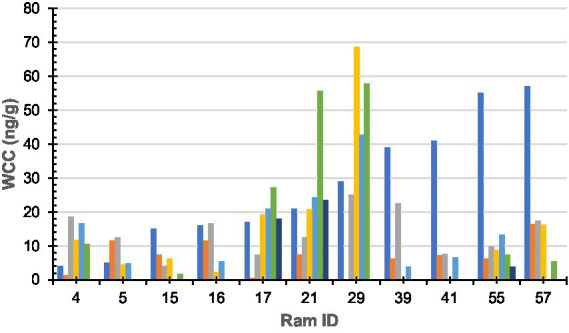
Wool cortisol concentration by 10-mm segment of sub-sample wool staple taken from rams (*n* = 11). Colours denote each segment of sub-sample wool (dark blue-a; orange-b; grey-c; yellow-d; light blue-e; green-f; and dark green-g). Ram sub-samples or 10-mm sections were as follows: Ram#4 = 5, Ram#5 = 4; Ram#15 = 4; Ram#16 = 4; Ram#17 = 6; Ram#21 = 6; Ram#29 = 4; Ram#39 = 4; Ram#41 = 3; Ram#55 = 6; Ram#57 = 4; and Ram#70 was removed due to contamination.

Cortisol concentration was determined for 11 rams with each 10-mm sample that was obtained and extracted (a total of 50 wool sub-samples had testosterone measured; and the number of sub-samples per ram is shown in the legend of Figure 1) using the DetectX^®^ Cortisol Immunoassay kit, manufactured by Arbor Assays. Briefly, a pipette was used to extract 500 μL of each sample into new, labelled Eppendorf tubes. The tubes were placed in a fume hood with the caps open to dry overnight. The remaining methanol sample was kept in the refrigerator for any future work. After drying, the samples were reconstituted with 25 μL of 100% methanol and 475 μL of ELISA assay buffer, which was prepared by diluting the assay buffer concentration provided in the DetectX^®^ Cortisol Immunoassay kit with distilled water in a ratio of 1:5.

Nunc™ 96-well plates were coated with 50 μL of cortisol antibody solution and incubated for at least 12 h. Standards were prepared serially using 200 μL of standard stock and 200 μL of assay buffer. Cortisol standards were run in duplicate from 400, 200, 100, 50, 25, 12.5, 6.25, 3.12, and 1.56 pg./well. Four non-specific binding (NSB) wells were used and included 75 μL of assay buffer was used and 50 μL of assay buffer to the two maximum binding wells (labelled “0” on the plate map. A volume of 25 μL of DetectX^®^ Cortisol conjugate was added to every well along with 25 μL of DetectX^®^ Cortisol antibody, which was omitted from the NSB wells. Once loaded, the plate was covered using sealing film, labelled with the assay type and time, and set atop the plate shaker at 900 rpm for 1 h. Next, the plate was washed four times using an automatic plate washer. Once washed, the plate was gently tapped dry using a paper towel. After adding 100 μL of tetramethylbenzidine (TMB substrate) to each well, the plate was resealed and allowed to incubate at room temperature for 30 min. Then, 50 μL of stop solution was added to each well. The plate was read at 450 nm using a microplate reader. Cortisol concentrations were exported as a CSV. Data were transformed from absorbance values (AU cm-1) into cortisol concentrations provided in nanograms per gram (ng/g).

### Testosterone assay

**Figure 2:**
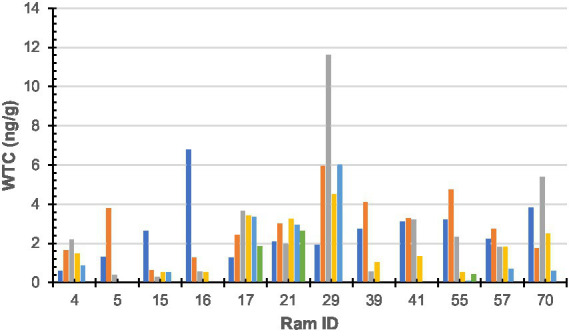
Wool testosterone concentration by 10-mm segment of sub-sample wool staple taken from rams (*n* = 12). Colours denote each segment of sub-sample wool (dark blue-a; orange-b; grey-c; yellow-d; light blue-e; green-f; and dark green-g). Ram sub-samples or 10-mm sections were as follows: Ram#4 = 5, Ram#5 = 3; Ram#15 = 5; Ram#16 = 4; Ram#17 = 6; Ram#21 = 6; Ram#29 = 5; Ram#39 = 4; Ram#41 = 4; Ram#55 = 6; Ram#57 = 5; and Ram#70 = 5.

Testosterone concentration was determined in 12 rams (a total of 53 wool sub-samples had testosterone measured as indicated in Figure 2) using the R156/7 enzyme within an immunoassay. A 96-well Nunc™ Maxisorp plate was coated the day prior to the assay. A volume of 25 μL of antibody stock was added to 6,225 μL of coating buffer at a working dilution of 1:25,000. The first column was reserved as non-specific binding wells and was coated with a coating buffer and without an antibody. Then, 50 μL of antibody was added per well using a multi-pipette. The plate was gently tapped on the table to maximise antibody coverage, covered and left for at least 12 h at 4°C.

Standards, including zeros and NSB, were prepared the following morning. Standard values were run in duplicate from 100, 50, 25, 12.5, 6.25, 3.12, 1.56, 0.78, and 0.39 pg./well. The standard working stock was diluted serially two-fold using 200 μL of stock and 200 μL of EIA buffer. This was repeated for all the remaining standards. The samples were diluted in EIA buffer. Then, 15 μL of testosterone conjugate [horseradish peroxidase (HRP)] was added to 5,985 μL of EIA buffer to create the working dilution.

An automatic plate washer was used to wash the plate four times with a wash solution. A paper towel was used to gently dry any remaining wash solution from the plate after washing. Using the plate map as a guide, 50 μL of standard, control, and sample were each added per well. Then, 50 μL of diluted testosterone HRP was added to all wells that contained standard, control, or sample. Within 10 min of beginning, the loaded plate was covered and left to incubate at room temperature for 2 h. Immediately after this time, the plate was washed four times with a wash solution using the automatic plate washer. The plate was briefly inverted to remove excess solution and carefully dried using a paper towel.

A volume of 50 μL of TMB substrate was added to wells that contained standard, control, or sample. The plate was covered with adhesive film and left to shake for 30 min for maximum colour development. A measure of 50 μL of stop solution was added to wells that contained standard, control, or sample. The plate was inserted into the plate reader and read at 450 nm. Absorbance values were exported as a CSV, imported into Microsoft Excel, and transformed to testosterone concentration (ng/g).

## Statistical analysis

Cortisol and testosterone absorbance data (AU cm^−1^) were provided by the plate reader as a CSV, which was transformed into both cortisol and testosterone concentration (ng/g) in Microsoft Excel. The first analysis included descriptive statistics, including mean, standard deviation, and standard error. A few outlier values were found. All such values were removed from all calculations. This included samples (10-mm sections) for cortisol analysis in 5E, 15D, 41C, 57D, 29A, and 70D due to random error. The samples (10-mm sections) removed from testosterone analysis included 5E and 5D due to random error. Wool hormone levels were compared within sub-samples and between individuals using a one-way ANOVA. All hormone data were log-transformed prior to analysis. Regression was performed to determine the relationship between wool testosterone and wool cortisol. A *p*-value of <0.05 represented levels of significance.

## Results

### Wool cortisol

Raw WCC for each ram is presented in Figure 1. No statistically significant (*F* = 0.93, df = 5, 44, *p* = 0.46) difference was found between an individual ram’s sub-sample; however, a statistically significant (*F* = 2.27, df = 10, 39, *p* = 0.03) difference was found between individual rams.

### Wool testosterone

No statistically significant (*F* = 1.33, df = 5, 52, *p* = 0.26) difference was found between an individual’s samples; however, a statistically significant (*F* = 2.17, df = 11, 46, *p* = 0.03) difference was found between individuals. Raw WTC for each sheep is presented in Figure 2.

### Wool cortisol and testosterone

**Figure 3:**
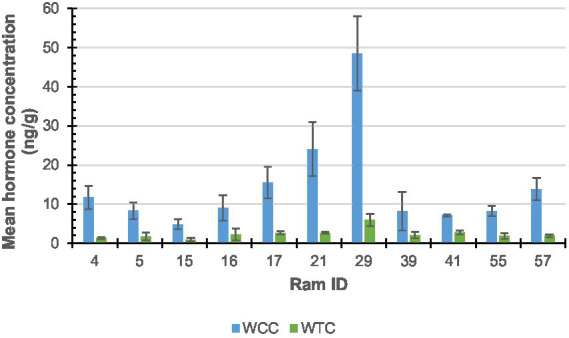
Ram wool mean cortisol (WCC; blue) and testosterone (WTC; green) concentrations.

As shown in Figure 3, the X-axis represents the ram with both cortisol and testosterone results (*n* = 11; notably, ram #70 was removed from cortisol analysis) identification number and the y-axis represents the hormone concentration, measured in ng/g. Error bars depict the standard error (SE) of each data set, which measures variability around the mean.

**Figure 4:**
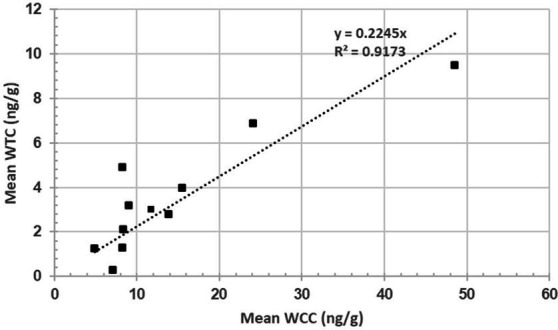
Relationship between wool cortisol concentration (WCC; ng/g) and wool testosterone concentration (WTC; ng/g).

The scatter plot represents the mean WCC (*n* = 11) and mean WTC (*n* = 11) for rams #4, 5, 15, 16, 17, 21, 29, 39, 41, 55, and 57. The linear regression intercept line is determined by the equation y = 0.2245x, suggesting WTC increases by 0.2245 ng/g per ng/g of WTC on average. The R^2^ of 0.9173 suggests a very strong, positive correlation (*p* = 3.65995E-05) between both hormones (Figure 4).

## Discussion

### Wool hormone assessment

This study assessed the variation in wool cortisol concentration (WCC) and wool testosterone concentration (WTC) along the length of the staple. No statistical difference (*p* > 0.05) in either wool cortisol or testosterone was found along the length of the staple. These findings are consistent with the study by Hantzopoulou et al. (20), who reported no statistically significant difference in the wool cortisol concentration when sub-sampling Merino ewe topknot fleece. Similarly, Davenport et al. (4) found no significant difference (*p* > 0.05) in cortisol between the distal and proximal portions of monkey hair. These results are replicated in a human hair study by Yang et al. (21), who found no significant difference in hair cortisol at three different lengths. Caution, however, must be applied during inter-species comparisons as several biological differences are at play. For instance, the use of human hair products is known to alter the hormone profile of hair. Nonetheless, it demonstrates that—to date—the assessment of hair segments is yet to yield a statistically significant difference, at least under mild stressors.

The present study did find a statistically significant difference (*p* < 0.05) in WCC and WTC between individual rams, demonstrating that the hormonal activity of individual rams varies within the flock. Hormones are chemical secretions that facilitate communication between bodily systems. Cortisol is the primary hormone of the mammalian stress response and is released by the adrenal glands, where it enters the bloodstream. Although the exact mechanism of incorporation is disputed, strong evidence supports the passive diffusion of hormones into the hair shaft through blood (1). As such, wool hormone concentration varies from animal to animal as a direct reflection of the level of HPA axis activity.

Our hypothesis, which suggested that there is no statistically significant difference in both wool cortisol and testosterone along the length of the staple, was proven correct. Additionally, we also confirmed that wool hormone concentrations vary significantly between individuals. As the rams were cohabitating under similar environmental conditions and much effort was taken to eliminate potential stressors, it is reasonable to expect similar hormone results, as each animal was experiencing comparable levels of HPA axis activity.

### Wool cortisol and testosterone relationship

Within many mammalian animal breeding populations, males actively compete against rival males in a dominance hierarchy for access to females. It is often the case that testosterone levels are related to the number of dominance disputes they have won and thus their position in the dominance hierarchy (22). Cortisol levels, however, vary across species, with examples of dominant males exhibiting low and higher blood glucocorticoid concentrations.

A positive association (R^2^ = 0.91) was found between WCC and WTC, which appears to be the first documented case of such a relationship in rams (Figure 4). A study by Medill et al. (22) found a non-significant, positive association (*p* > 0.05; R^2^ = 0.43) between hair cortisol and testosterone in feral horses. Bachelors (males of breeding age yet to win access to their own mare) were hypothesised to have lost more dominance disputes and thus have elevated cortisol levels (22). Although such a strong association is interesting—particularly in social animals, such as rams—it is difficult to confidently identify the exact reasoning for such a relationship from a single wool sample. Moreover, as wool samples were collected in October 2022, it is highly likely that seasonal changes that accompany the breeding season (February to June) are reflected in the samples and contribute to intra-sample hormone variability.

Future iterations of this study should first determine if seasonal hormone variability is reflected in wool, as in conventional blood samples. Second, in-field observations should be conducted to identify dominant rams within the flock, and later, repeat sampling and data analysis should be conducted to determine how wool cortisol and testosterone change alongside shifts in ram dominance hierarchy positioning.

## Conclusion

Wool could provide a suitable, non-invasive biological matrix for the assessment of ram cortisol and testosterone, avoiding the need for blood collection or faecal sampling. No statistically significant differences were found along the length of the staple for either hormone. These findings were confirmed by the literature. As hypothesised, a statistically significant difference was found between individuals. Measuring wool cortisol and testosterone provides an indication of the activity of the HPA and male HPG axes. Moreover, a strong correlation was found between wool cortisol and testosterone concentrations. After an extensive literature search, it is believed this study is the first to find such an association between reproductive and stress hormones in wool.

## Data availability statement

The original contributions presented in the study are included in the article. Further inquiries can be directed to the corresponding author.

## Ethics statement

The animal study was approved by Animal Ethics Committee—The University of Queensland. The study was conducted in accordance with the local legislation and institutional requirements.

## Author contributions

DF: Data curation, Formal analysis, Investigation, Methodology, Software, Visualization, Writing – original draft, Writing – review & editing. BW: Resources, Writing – original draft, Writing – review & editing. EN: Conceptualization, Data curation, Formal analysis, Funding acquisition, Investigation, Methodology, Project administration, Resources, Software, Supervision, Validation, Visualization, Writing – original draft, Writing – review & editing.

## References

[1] Russell E Koren G Rieder M Van Uum S. Hair cortisol as a biological marker of chronic stress: current status, future directions and unanswered questions. Psychoneuroendocrinology. (2012) 37:589–601. doi: 10.1016/j.psyneuen.2011.09.009 21974976

[2] Salaberger T Millard M El Makarem S Möstl E Grünberger V Krametter-Frötscher R. Influence of external factors on hair cortisol concentrations. Gen Comp Endocrinol. (2016) 233:73–8. doi: 10.1016/j.ygcen.2016.05.005, PMID: 27167500

[3] Sejian V Bhatta R Gaughan J Dunshea F Lacetera N. Adaptation of animals to heat stress. Animal. (2018) 12:s431–44. doi: 10.1017/S1751731118001945 30139399

[4] Davenport MD Tiefenbacher S Lutz CK Novak MA Meyer JS. Analysis of endogenous cortisol concentrations in the hair of rhesus macaques. Gen Comp Endocrinol. (2006) 147:255–61. doi: 10.1016/j.ygcen.2006.01.005, PMID: 16483573

[5] Cook NJ. Minimally invasive sampling media and the measurement of corticosteroids as biomarkers of stress in animals. Can J Anim Sci. (2012) 92:227–59. doi: 10.4141/cjas2012-045

[6] Hamilton TRDS Mendes CM Castro LSD Assis PMD Siqueira AFP Delgado JDC. Evaluation of lasting effects of heat stress on sperm profile and oxidative status of ram semen and Epididymal sperm. Oxidative Med Cell Longev. (2016) 2016:1687612. doi: 10.1155/2016/1687657 PMC4737001 26881013

[7] Thibault C. Levasseur M.-C. (2001). Reproduction in mammals and man. INRA Editions. Ellipses.

[8] Perkins A Roselli CE. The ram as a model for behavioral neuroendocrinology. Horm Behav. (2007) 52:70–7. doi: 10.1016/j.yhbeh.2007.03.016, PMID: 17482616 PMC2150593

[9] Schanbacher B Crouse J Ferrell C. Testosterone influences on growth, performance, carcass characteristics and composition of young market lambs. J Anim Sci. (1980) 51:685–91. doi: 10.2527/jas1980.513685x, PMID: 7440452

[10] Walker D. The influence of sex upon carcass quality of New Zealand fat lamb. New Zealand J Sci Technol. (1950) 32:30–8. doi: 10.5555/19511402860

[11] Thwaites CJ. Development of mating behaviour in the prepubertal ram. Anim Behav. (1982) 30:1053–1059.

[12] Cobb T Hantzopoulou G-C Narayan E. Relationship between wool cortisol, wool quality indices of Australian merino rams and climatic variables in Tasmania. Front Anim Sci. (2023) 4:1234343. doi: 10.3389/fanim.2023.1234343

[13] Narayan E Sawyer G Fox D Smith R Tilbrook A. Interplay between stress and reproduction: novel epigenetic markers in response to shearing patterns in Australian merino sheep (*Ovis aries*). Front. Vet. Sci. (2022) 9:830450. doi: 10.3389/fvets.2022.830450, PMID: 35464367 PMC9021797

[14] Morrow C. Kolver E. Verkerk G. Matthews L. (2000). Urinary corticosteroids: an indicator of stress in dairy cattle. Proceedings-New Zealand Society of Animal Production.

[15] Nejad JG Lohakare J Son J Kwon E West J Sung K. Wool cortisol is a better indicator of stress than blood cortisol in ewes exposed to heat stress and water restriction. Animal. (2014) 8:128–32. doi: 10.1017/S1751731113001870, PMID: 24182313

[16] Nejad JG Sung K Lee B Peng J Kim J Oh S. Effects of adding water to total mixed ration on water consumption, nutrient digestibility, wool cortisol, and blood indices in Corriedale ewes under hot and humid conditions. J Anim Sci. (2016) 94:832–2. doi: 10.2527/jam2016-1707

[17] Sawyer G Fox DR Narayan E. Pre-and post-partum variation in wool cortisol and wool micron in Australian merino ewe sheep (*Ovis aries*). PeerJ. (2021) 9:e11288. doi: 10.7717/peerj.11288, PMID: 33987000 PMC8086564

[18] Weaver SJ Hynd PI Ralph CR Edwards JH Burnard C Narayan E. Chronic elevation of plasma cortisol causes differential expression of predominating glucocorticoid in plasma, saliva, fecal, and wool matrices in sheep. Domest Anim Endocrinol. (2021) 74:106503. doi: 10.1016/j.domaniend.2020.106503, PMID: 32846373

[19] Sawyer G Webster D Narayan E. Measuring wool cortisol and progesterone levels in breeding maiden Australian merino sheep (Ovis aries). PLoS One. (2019) 14:e0214734. 30958853 10.1371/journal.pone.0214734 PMC6453452

[20] Hantzopoulou GC Sawyer G Tilbrook A Narayan E. Intra-and inter-sample variation in wool cortisol concentrations of Australian merino lambs between twice or single shorn ewes. Front Anim Sci. (2022) 3:890914.

[21] Yang HZ Lan J Meng YJ Wan XJ Han DW. A preliminary study of steroid reproductive hormones in human hair. J Steroid Biochem Mol Biol. (1998) 67:447–50. doi: 10.1016/S0960-0760(98)00120-4, PMID: 10030694

[22] Medill SA Janz DM McLoughlin PD. Hair cortisol and testosterone concentrations in relation to maturity and breeding status of male feral horses. Animals. (2023) 13:2129. doi: 10.3390/ani13132129, PMID: 37443926 PMC10339860

